# Superficial thrombophlebitis (Mondor's Disease) after breast augmentation surgery

**DOI:** 10.4103/0970-0358.44940

**Published:** 2008

**Authors:** Giovanni André P. Viana, Fabrício M. Okano

**Affiliations:** 1 Member of Brazilian Plastic Surgery Society, Member of International College of Surgeons, Brazil; 2 Vascular Surgeon. Brazil

**Keywords:** Diagnosis, mammoplasty, Mondor's disease, superficial thrombophlebitis, surgery

## Abstract

Although the aetiology of Mondor's disease remains unclear, the most commonly cited cause is trauma of some sort. Although surgical trauma has frequently been quoted, reports that specifically implicate aesthetic breast surgery are unusual in the literature. In this article, the authors report a case of superficial thrombophlebitis of the anterolateral chest wall secondary to breast augmentation surgery in a woman, five months after the procedure. The authors performed an analysis of the disease's main etiologic components and preponderant clinical aspects, and determined all appropriate therapeutic measures.

Mondor's disease is characterized by superficial thrombophlebitis or phlebitis of the anterior or lateral wall of the chest involving the lateral thoracic, thoracoepigastric, or superior epigastric veins.[1–4] About 75% of the cases are women, generally between the second and fifth decades of life. Most cases are unilateral, and the most commonly affected vessel is the thoracoepigastric vein.[1–6]

The authors present here a case of superficial thrombophlebitis of the left anterolateral chest wall in a young female who had undergone breast augmentation five months prior to the appearance of the initial clinical symptoms. The authors also analysed the main etiologic components and preponderant clinical aspects of the disease, and determined all appropriate therapeutic measures.

## CASE REPORT

**Figure 1:**
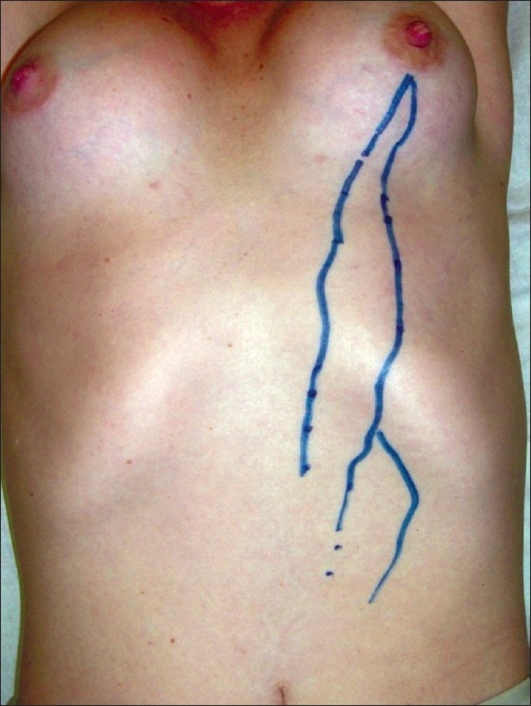
The three curvilinear lesions

**Figure 2:**
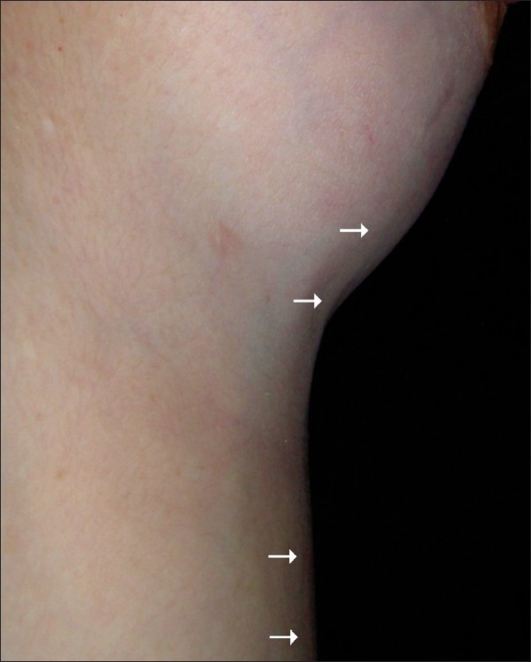
Lateral view

**Figure 3:**
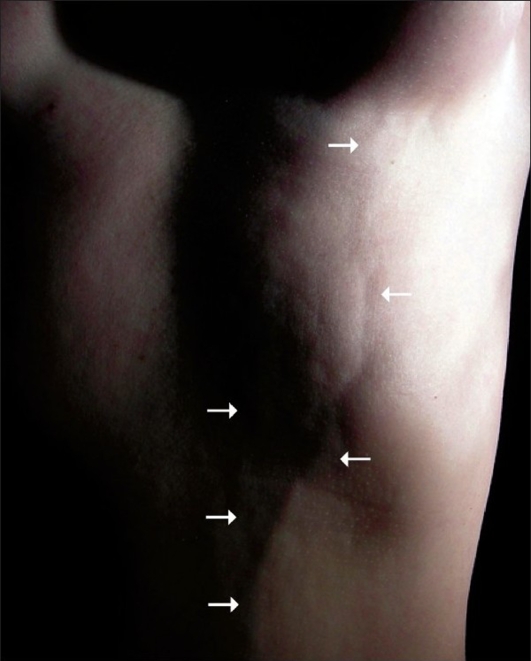
Front view

The case is of R.C.T.O., a 33 year-old female, who presented with hypomastia. The patient underwent breast augmentation through inferior periareolar incision with round silicone gel breast implants (high-strength, silicone gel-filled, polyurethane foam-covered) in February 2006. The implants were placed in the retromammary location above the pectoralis muscle. The postoperative course was entirely uneventful until five months later, when she sought advice because she had been complaining of a moderate burning pain in the left anterolateral thoracic region for five days, which worsened upon abducting the left shoulder. Three days later, she noted the appearance of three curvilinear lesions running vertically from the inferior pole of the left breast to the umbilicus [Figures 1–3]. No other symptoms were reported.

The patient denied any previous use of drugs, trauma, or other pathologies. She was not obese (body mass index of 22 kg/m^2^), was a non-smoker, had no previous history of thromboembolic disease, and had been using oral contraception for three years (desogestrel 25 *µ*g; ethinyl estradiol 40 *µ*g). However, her gynaecologist had changed her medication two months before the initial symptoms (desogestrel 15 *µ*g; ethinyl estradiol 20 *µ*g). The physical examination revealed that the arterial pressure was 110 / 70 mm Hg, the heart rate was 78 beats per minute, and the respiratory frequency was 18 cycles per minute. The presence of three curvilinear cords in the anterolateral chest wall was noted, the bigger one extended down from the inferior-medial pole of the left breast onto the umbilicus. The two remaining cords were smaller and didn't have a precise origin. However, the three cords were adherent to the skin and subcutaneous tissue; they were slightly painful when touched but presented no phlogistic signs, and corresponded to the topography of drainage of the left thoracoepigastric vein and its branches. The breasts were symmetrical and the nipple-areola complex was normal. The head, neck, breasts, lungs, precordium, abdomen, and extremities remained unaltered and the general condition of the patient was unaffected.

Although the clinical diagnosis of Mondor's disease was clear, complementary tests were performed to rule out other pathologies.[2–7] The results of the laboratory investigation (including hemogram, serum lipids, liver function tests, D-dimer, erythrocyte sedimentation rate, C-reactive protein, autoantibody screening, blood clotting levels, and thrombophylia screening) were normal. Breast and axillary ultrasound evidenced thrombophlebitis of the left thoracoepigastric vein and ruled out the possibility of breast cancer.

The patient underwent oral therapy with nonsteroidal anti-inflammatory drugs and her course of oral contraception was interrupted. Low molecular weight heparin (LMWH) was given subcutaneously (20 mg/day) for five days and a topical analgesic with heparinoid cream was used. She presented a progressive regression of painful symptoms in the subsequent week and the lesions disappeared two weeks later.

## DISCUSSION

Classical Mondor's disease affects veins that protrude from the mammary nipple-areolar complex and axilla (lateral thoracic vein), above and around the epigastrium (superior epigastric vein) and in the costal margin and superior abdominal wall (thoracoepigastric vein).[1–6] This filiform phlebitis occurs at the same frequency in both sides of the thorax and most cases are unilateral.[1] The typical presentation is that of an abrupt onset of pain in the breast or chest wall, followed by the appearance of a firm, red, tender cord corresponding to the location of one of those three superficial chest wall veins.[1–2] Typically, the course of the disease is self-limiting, lasting weeks or rarely months, before resolving spontaneously without any risk of embolisation.[1–7]

The aetiology remains an object of speculation, but it has been associated more often with local trauma such as surgical biopsy, breast surgery; mammary inflammations and infections; breast cancer, large and pendulous breasts, local muscle strains, vigorous upper extremity exercise, tight clothes, abusive use of intravenous drugs, or even an association of these factors.[1–4]

A diagnosis of Mondor's disease can be made satisfactorily on the basis of clinical history and findings. However, in cases when the aetiology is not defined, patients should undergo a rigorous diagnostic investigation to determine the cause—a hidden breast cancer or lymphatic spread from carcinoma, a hypercoagulability state or a connective tissue disease.[2,3,7,8]

Conservative therapy includes local application of heat, use of systemic or topical anti-inflammatory medication, and general measures such as support for the assailed breast.[1–3,7] Anticoagulation therapy is not normally necessary and antibiotics are not required unless there is evidence of documented infection.[1–3,7] Locally acting anticoagulants/antithrombotics have a positive effect on both pain as well as reduction of the size of the thrombus and systemic oral anticoagulation is rarely requested.[7,9]

Nevertheless, the American College of Chest Physicians and several other consensus documents suggest using low molecular weight heparin (LMWH) to prevent thrombosis in subjects at higher risk, mainly in patients with primary superficial thrombosis, which may be associated with other more severe underlying diseases.[7,9,10] Following this guideline, the authors considered prescribing in this case, both low molecular weight heparin (Enoxaparin 20 mg/day for five days) and topically applied anticoagulants/antithrombotics in the acute phase while complete laboratory and radiological investigations were conducted. These drugs appear effective in terms of reducing local pain, swelling and redness, increasing the patient's mobility, and in preventing local thrombus growth.[7,9]

The subject in this case presented a progressive regression of painful symptoms in the subsequent week, and returned to her daily chores immediately. The cord lesions disappeared two weeks after appropriate therapeutics were begun. An year and a half after the diagnosis of Mondor's disease, she has been asymptomatic with no other thromboembolic/thrombophlebitis events.
